# A retrospective review of methylamphetamine detected in child deaths reported to the Victorian Coroner, Australia

**DOI:** 10.1007/s12024-024-00778-8

**Published:** 2024-01-19

**Authors:** Dylan Mantinieks, Sarah Parsons, Jennifer Schumann, Olaf H. Drummer, Kerryn Crump, Yeliena Baber, Melanie Archer, Dimitri Gerostamoulos

**Affiliations:** 1 Department of Forensic Medicine, School of Public Health and Preventive Medicine, Monash University, 65 Kavanagh Street, Southbank, Victoria 3006 Australia; 2 Victorian Institute of Forensic Medicine, 65 Kavanagh Street, Southbank, Victoria 3006 Australia

**Keywords:** Child mortality, Forensic toxicology, Hair analysis, Amphetamines, Methylamphetamine

## Abstract

This study investigated methylamphetamine (MA) exposures in the deaths of children (≤ 12 years old) reported to the Coroner in the state of Victoria, Australia, between 2011 and 2020. Demographics, autopsy findings including the cause of death, self-reported prenatal or caregiver drug use, child protection services information, and toxicological findings were summarized by descriptive statistics. Validated methods of liquid chromatography-tandem mass spectrometry were used in the analysis of drugs. There were 50 child deaths with MA detected in blood, urine, and/or hair with 64% (*n* = 32) identified in 2018–2020. Most children were 1–365 days old (66%, *n* = 33) and the cause of death was unascertained in 62% (*n* = 31) of cases. MA was toxicologically confirmed in hair (94%, *n* = 47) significantly more than blood (18%, *n* = 9). Prenatal or caregiver drug use was self-reported in 44% (*n* = 22) and 42% (*n* = 21) of cases, respectively. Moreover, only 54% (*n* = 27) of deceased children were a child protection client at their time of death. These findings suggest the number of deceased children exposed to MA has increased over the past 10 years, which is consistent with the greater supply of crystal MA in the Australian community. Hair analysis provided additional means to identify cases that were unknown to child protection services and may have implications for other children in the same drug exposure environment.

## Introduction

Methylamphetamine (MA) is an amphetamine-type stimulant that elicits an intense euphoria and stimulation of the central nervous system [1, 2]. The most common routes of administration of MA include smoking and intravenous injection due to their rapid onset of effects [3]. Amphetamine is the primary metabolite of MA but is also prescribed for attention deficit hyperactive disorder in children [4]. General population surveys suggest recent amphetamine-type stimulant use (past 12 months use) in Australia has decreased in the past 10 years from 2.2% in 2010 to 1.4% in 2019 [5]. However, rises in the global supply of crystal MA have been associated with increases in MA-related harms including deaths involving MA [6, 7].

Prenatal and postnatal exposure to some illicit drugs is associated with an increased risk of sudden infant death syndrome (SIDS) and sudden unexplained death in children older than 1 year [8]. Studies examining the risk of death among children exposed to MA are limited, but may contribute to a generic increased risk of SIDS similar to other stimulants like cocaine [9]. Kandall et al. found that the rates of SIDS in drug-exposed and non-drug-exposed infants were 5.83 and 1.39 per 1000 infants, respectively [10]. Similarly, Ward et al. found the incidence of SIDS was higher in the infants born to substance-abusing mothers compared to the general population [11].

Most drugs in blood and urine may be detected for hours to days, while hair may represent many months of drug exposure depending on the length of the hair fiber [12, 13]. Therefore, hair analysis offers the longest window of detection to identify previous MA exposure [14]. The detection of MA in the hair of children may be attributed to (1) exposure *in utero* or through breastmilk secondary to maternal use; (2) accidental consumption by the child or deliberate administration by the caregiver; or (3) environmental contamination including but not limited to exposure to secondhand smoke or close contact with caregivers using MA [15]. Thirdhand smoke (i.e., household surfaces contaminated with MA from previous manufacture or use) may also represent another way MA deposits in hair [16]. For example, hair analysis was able to confirm MA exposure in 45–73% of children removed from clandestine laboratories [17, 18]. However, distinguishing between systemic and environmental exposure is limited due to the ineffectiveness of decontamination procedures to wash the hair and the lack of MA metabolites as markers of consumption [19–21].

This study aimed to investigate child deaths with MA detections between 2011 and 2020 to determine if the number of exposures to MA in these deaths has increased over time.

## Methods

### Data sources

Data on child deaths involving MA were obtained from the Victorian Institute of Forensic Medicine (VIFM) Internal Case Management System database. The VIFM is a centralized independent statutory authority in Victoria, Australia, that assists in the investigation of > 7000 deaths per annum reported to the Victorian State Coroner by providing forensic medical and scientific services. The VIFM database was searched for deaths with MA detected in blood, urine, and/or hair between 1 January 2011 and 31 December 2020.

Data over the same time was also sourced from the National Coronial Information System (NCIS) database, a storage and retrieval system for deaths reported to Australian and New Zealand Coroners. The Victorian Department of Justice and Community Safety was the organizational source of the data. “Methylamphetamine” was selected from the Pharmaceutical Substance for Human Use drug search function to identify closed coronial cases in which MA was included in the cause of death, determined to have contributed to death, or another MA affected person was a factor in the death [22]. Age was restricted to ≤ 12 years old, the jurisdiction was Victoria only, and the study period related to notifications during the study period. This additional search strategy on the national database ensured that all relevant cases from the study period were captured.

### Case inclusion/exclusion

The cohort comprised of child deaths (≤ 12 years old) reported to the Coroner in the state of Victoria, Australia, and MA was detected in blood, urine, and/or hair. Neonates, infants, and children (≤ 12 years old) were included and stratified into age groups (< 1 day old, 1–365 days old, and > 1 year old). Adolescents (> 12 years old) and adults were excluded.

### Medico-legal death investigation

In Victoria, medico-legal death investigations conducted by forensic pathologists may involve external inspection and/or internal examination at autopsy authorized by the Coroner. This may involve the submission of an objection to internal examination by the senior next-of-kin based on religious, cultural, or other reasons — these are usually granted. Internal examination at autopsy typically involves an investigation of the major organ systems by macroscopy and histology, including neuropathology, with ancillary tests (e.g., microbiology, metabolic screen, biochemistry, and toxicology). In addition, all deceased children admitted to the mortuary had a computerized tomography scan (+/− skeletal survey) with a report prepared by a pediatric radiologist.

Specimens for toxicological analysis were collected at mortuary admission and/or autopsy with pathologists’ approval. Post-mortem femoral blood was collected by upper thigh venous puncture as soon as practicable after mortuary admission, except children < 2 years old. Additional post-mortem blood, urine, hair, and other tissue specimens may have been collected at autopsy. Post-mortem blood specimens were preserved with 1% w/v sodium fluoride and potassium oxalate and stored at 4 °C until analysis. If the deceased child had been in hospital, ante-mortem (AM) toxicology specimens were requested and stored at −20 °C. Hair from was cut from the vertex posterior prior to internal examination at autopsy, although in some cases this may have been collected from other head regions to obtain sufficient hair mass. Dry hair specimens were secured in foil with the root end identified and stored at room temperature away from direct sunlight.

### Toxicological analysis

Previously published and validated methods of liquid chromatography-tandem mass spectrometry (LC–MS/MS) were used to detect common and toxicologically relevant acidic, basic, and neutral drugs in blood [23, 24]. Urine and hair were also analyzed using validated LC–MS/MS methods [25, 26]. It is noteworthy that the analysis for toxicologically significant drugs in blood is routine, and the analytical method sensitivity for MA did not change between 2011 and 2020. However, hair analysis is requested at the discretion of the investigating pathologist often prompted by trace detections of drugs in routine blood screening (i.e., concentrations less than limits of reporting). The limits of reporting for MA in blood, urine, and hair were ≥ 0.02 mg/L, ≥ 0.05 mg/L, and ≥ 0.02 ng/mg, respectively.

### Data collection

Pathology reports, police findings of circumstances, and Coroners’ findings were reviewed. Variables included the year of death, age, sex, autopsy type (external inspection or internal examination), and cause of death. The cause and manner of death were classified according to Table 1. Group A comprised complications of prenatal drug use including placental hemorrhage, intrauterine growth restriction, and premature delivery. Other drug use during pregnancy could not be excluded in group A cases. Also, prenatal drug use was not necessarily excluded in groups B–D, though it was not opined to have contributed to death. The Sleep-Related Sudden Unexpected Death of an Infant or Child Investigative Checklist that was completed in the deaths of young children by police as part of the Brief of Evidence for the Coroner was reviewed. The checklist included sibling(s) information, breastfeeding, drug exposure, and scene findings of drug, tobacco, and alcohol use. The involvement of child protection services (CPS) specified in the notification of child death responses was also reviewed. CPS notified of the death of a child are required to inform the Coroner if that child was a CPS client and to provide details of their involvement.

**Table 1:** Classification of the cause of death

Group	Cause of death	Description
A	Complications of prenatal drug use	Deaths due to complications of maternal drug use during pregnancy
B1	Unascertained without co-sleeping	Unexplained deaths where co-sleeping was excluded
B2	Unascertained with co-sleeping	Unexplained deaths with evidence of co-sleeping
B3	Unascertained with other complicating factors	Unexplained deaths in the setting of other factors (e.g., unexplained trauma or drugs in blood/urine)
B4	Unascertained without internal examination	Unexplained deaths limited to external inspection
C	Natural causes	Deaths due to natural causes (e.g., asthma or infection)
D	Other causes	External or other causes of death not appropriately classified into groups A–C

Toxicological findings were extracted from toxicology reports or the VIFM Internal Case Management System. MA detected in hair with insufficient mass (< 20 mg) and wash solutions are presented as qualitative determinations only. Analytical hair preparation records provided information on the hair length analyzed and hair color. Also, other drugs detected were grouped into other amphetamine-type stimulant excluding MA, anesthetics, analgesics, anti-convulsants, anti-depressants, anti-histamines, asthmatic drugs, benzodiazepines, cannabinoids, cardiovascular drugs, cocaine, heroin, opioid narcotics, other stimulants and anorectics, and other substances classed as drugs.

### Statistics

The presented study is descriptive only. Continuous variables were summarized using means or medians, and categorical variables as percentages with low frequencies presented as < 5.

### Ethics

The study was approved by the VIFM Ethics Committee (1188–1180/1), Coroners Court of Victoria Research Committee (RC 408), and the Department of Justice Human Research Ethics Committee (CF/21/10851).

## Results

### Demographics and cause of death

**Fig. 1:**
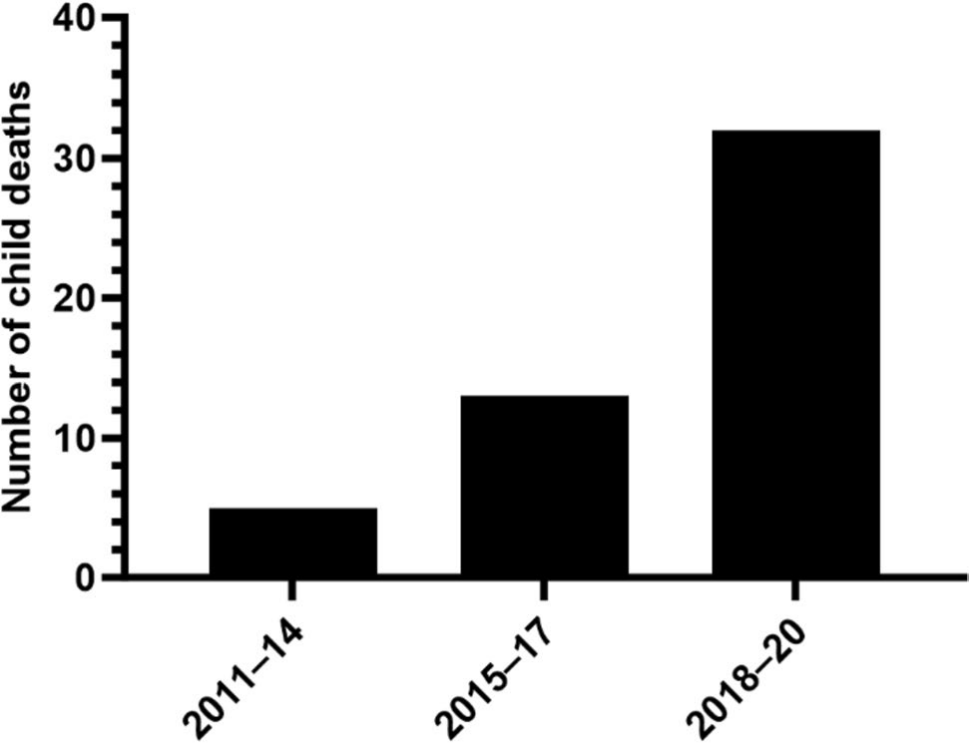
Number of child deaths in which MA was detected between 2011 and 2020 (*n* = 50)

There were 50 child deaths in which MA was detected in blood, urine, and/or hair between 2011 and 2020. Figure 1 shows the increased number of deaths over time, noting the low sample size, with 64% (*n* = 32) of deaths occurring between 2018 and 2020. Most deceased children were 1–365 days old (66%, *n* = 33) where the median age was 47 days old (range = 1–349) (see Table 2). Medico-legal death investigations involving internal examination or external inspection only comprised 88% (*n* = 44) and 12% (*n* = 6) of cases, respectively. The cause of death was classified as groups B1–4 in 62% (*n* = 31) of deaths, predominately group B2 and group B3, while group A deaths mostly involved known complications associated with prenatal MA use such as intrauterine growth restriction and premature delivery [27].

**Table 2:** Demographics and cause of death in child deaths in which MA was detected (*n* = 50)

	%	*n*	Median (range)
Age			
- < 1 day old	12	6	
- 1–365 days old	66	33	47 (1–349)
- > 1 year old	22	11	2.2 (1.2–8.7)
Sex			
- Male	62	31	
- Female	38	19	
Cause of death			
- Group A	14	7	
- Group B1	< 10	< 5	
- Group B2	24	12	
- Group B3	22	11	
- Group B4	10	5	
- Group C	14	7	
- Group D^*^	10	5	

*Other cause of death included perinatal asphyxia, trauma, immersion, and effects of fire

### Drug exposure information

Prenatal and caregiver drug use was self-reported in 44% (*n* = 22) and 42% (*n* = 21) of deaths, respectively (see Table 3). However, drug use was denied in 26% (*n* = 13) of pregnant women and 24% (*n* = 12) of caregivers. Breastfeeding, a potential route of exposure to maternal drug use, was reported in 42% (*n* = 21) of cases. Signs of drug, tobacco, and alcohol use were reported by attending police officers or paramedics in 24% (*n* = 12) of deaths, although the checklist was not always completed in the home environment. Importantly, about one in two deceased children (54%, *n* = 27) was known to CPS at the time of their death and 76% (*n* = 38) were known to have sibling(s).

**Table 3:** Drug exposure information and toxicological findings of MA

	%	*n*	Median (range)
Self-reported prenatal drug use			
- Yes	44	22	
- No	26	13	
- Unknown	30	15	
Self-reported caregiver drug use			
- Yes	42	21	
- No	24	12	
- Unknown	34	17	
MA concentrations in blood			
- Detected ≥ 0.02 mg/L	18	9	0.19 (0.022–0.99)
- Not detected < 0.02 mg/L	82	41	
- Amphetamine detected ≥ 0.02 mg/L	10	5	0.17 (0.15–0.46)
MA concentrations in urine			
- Detected ≥ 0.05 mg/L	< 10	< 5	0.13 (0.057–0.17)
- Not detected < 0.02 mg/L	92	46	
- Amphetamine detected ≥ 0.05 mg/L			
MA concentrations in hair			
- Detected ≥ 0.02 ng/mg	94	47	0.89 (0.026–10)*
- Not detected < 0.02 ng/mg	< 10	< 5	
- Detected in wash solution	42	21	
- Amphetamine detected ≥ 0.02 ng/mg	60	30	0.42 (0.030–2.4)^**^

*n = 14

^**^n = 10

### Toxicological findings in blood, urine, and hair

**Fig. 2:**
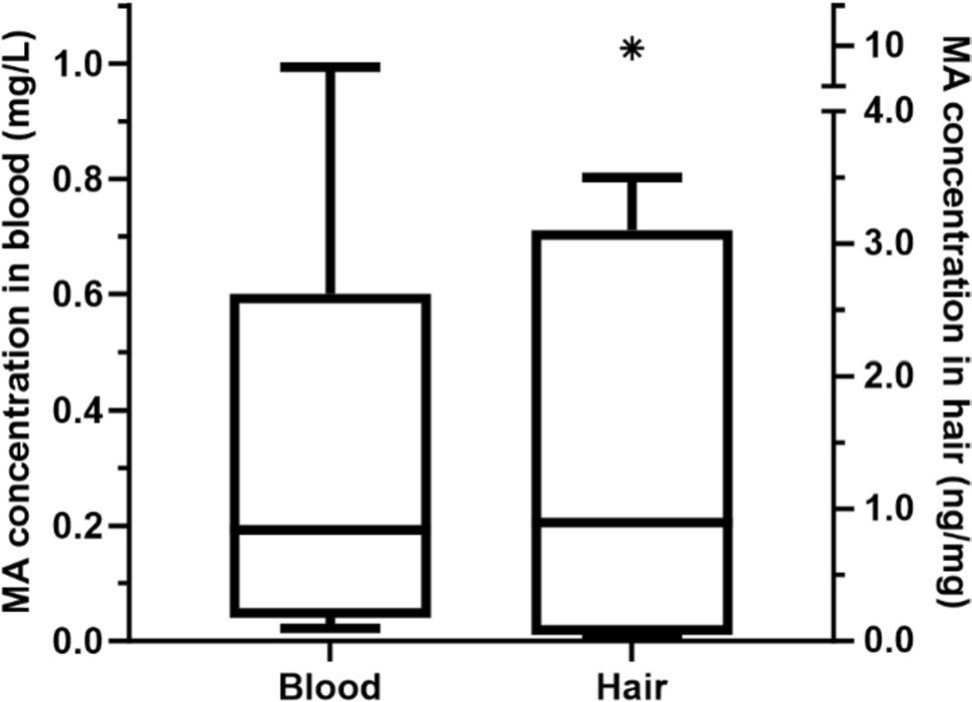
Box and whisker plots of MA concentrations in blood (*n* = 9) and hair (*n* = 14)

The toxicological findings of MA in blood, urine, and hair are presented in Table 3. Blood, urine, and hair were analyzed in 98% (*n* = 49), 30% (*n* = 15), and 94% (*n* = 47) of child deaths, respectively. Notably, urine was only available for analysis in 21% of children 1–365 days old (*n* = 7) compared to 73% of children > 1 year (*n* = 8). The source of post-mortem blood analyzed was femoral (18%, *n* = 9), subclavian (10%, *n* = 5), heart (38%, *n* = 19), cavity (16%, *n* = 8), or brain (< 10%, *n* < 5), while AM blood was analyzed in 14% (*n* = 7) of cases. MA was reported in blood in 18% (*n* = 9) of deaths with a median concentration of 0.19 mg/L (range = 0.022–0.99 mg/L). In hair, MA was reported as detected in 94% (*n* = 47) of cases, and Fig. 2 shows the dispersion of MA hair concentrations (median = 0.89 ng/mg, range = 0.026–10 ng/mg) in 14 deaths with sufficient hair mass (≥ 20 mg). Moreover, the wash solutions were positive for MA in 42% (*n* = 21) of deaths. The median concentration of amphetamine in hair was 0.42 ng/mg (range = 0.030–2.4, *n* = 10), and the amphetamine/MA ratio was 0.045–48 (median = 0.11, *n* = 10). There were only nine deaths where MA was the only drug detected; other drugs included other amphetamine-type stimulants excluding MA (12%, *n* = 6), anesthetics (14%, *n* = 7), analgesics (20%, *n* = 10), anti-convulsants (< 10%, *n* < 5), anti-depressants (< 10%, *n* < 5), anti-histamines (< 10%, *n* < 5), asthmatic drugs (< 10%, *n* < 5), benzodiazepines (24%, *n* = 12), cannabinoids (26%, *n* = 13), cardiovascular drugs (< 10%, *n* < 5), cocaine (20%, *n* = 10), heroin (12%, *n* = 6), opioid narcotics (56%, *n* = 28), simulants and anorectics (< 10%, *n* < 5), and other substances classed as drugs (< 10%, *n* < 5).

## Discussion

Toxicological findings in these deceased children demonstrate an increase in exposures to MA over the last decade, although its significance is uncertain. Indeed, many deaths were designated as unascertained (62%) since MA detected in hair was considered largely non-contributory to death. This is consistent with recommendations about the need for toxicological testing in unexplained child deaths or SIDS regardless of its low diagnostic yield in the determination of the cause of death [28]. These recommendations do not concern hair analysis for drugs despite its improved rates of detection relative to blood and urine. For instance, MA was confirmed only in hair in 76% of deceased children (i.e., MA was not reported in blood and urine). In the limited number of deaths in which MA was detected in blood, concentrations of MA overlapped with those previously reported in the literature. For example, the post-mortem blood concentrations were 0.03–1.2 mg/L (median = 0.35 mg/L) in eight fetal and infant deaths associated with MA, where only one death was attributed to the direct toxic effects of MA (MA concentration = 1.2 mg/L) [29]. Five of nine deaths with MA detected in blood in the present study were likely from *in utero* exposure to MA nearer parturition considering they were 0–1 days old and prenatal drug use was self-reported in all five cases, while breastfeeding was a more likely route of systemic exposure in the other four deaths.

The incorporation rates of drugs into hair correlate with their  physicochemical properties and melanin content [13]. Basic drugs (e.g., MA) avidly bind to eumelanin that constitutes the majority of melanin content in black and brown hair [13]. Furthermore, the structurally thinner and more porous hair of children may increase its susceptibility to secondhand or thirdhand contamination [15]. Hair concentrations of MA in children removed from clandestine laboratories (median = 7.0 ng/mg, range = 0.1–131 ng/mg, *n* = 52) were higher than those in children removed from home environments of alleged substance misuse (median = 1.4 ng/mg, range = 0.1–22 ng/mg, *n* = 67) [14, 18], while hair concentrations in four children exposed to thirdhand smoke from MA use were 0.007, 0.008, 0.013, and 0.040 ng/mg [16]. There were no deaths in the present study that occurred in the setting of MA manufacture, and hair concentrations appear to be more consistent with secondhand exposure to MA use.

Rises in the most overt MA-related harms (psychosis, dependence, and violence) have been attributed to the increased availability of crystal MA smoked in patterns of heavy binge use [6]. Indeed, smoking as the primary route of administration in people who recently used MA increased from 19.1% in 2010 to 41.1% in 2019 [5]. This is significant because pharmacokinetic studies demonstrate that MA readily accumulates in lung tissue, which is a recognized drug reservoir in post-mortem redistribution [30, 31]. It is possible that sequestered amounts in lung tissue from passive inhalation led to the post-mortem redistribution of MA. Consequently, trace concentrations of MA detected in routine blood screening prompted more specialized hair analysis where MA was detected at reportable concentrations.

The adverse health effects from breathing in secondhand smoke are unknown, although likely compounded by the concomitant exposure to other drugs like heroin and cocaine [32]. Wright et al. reported the adverse health effects associated with MA-contaminated properties (e.g., respiratory, skin, and eye irritation) resolved when children were removed from drug exposure environments [16]. What may be more significant is the association between caregiver substance use and child maltreatment (e.g., emotional, sexual, and physical abuse or neglect), which may have implications for other children in the same drug-using environment [33]. Opportunities to improve the outcomes for children at risk may also be missed considering 46% of deaths were not known to CPS, and local legislation only requires child death review committees to conduct inquiries into the deaths of children who were CPS clients at the time of death or 12 months prior to death [34]. Pragst et al. contrasted the hair analysis results of drug-using caregivers and their children in 140 families [35]. There was agreement in drug detections between caregivers and their children in 48% of families, and the same drugs were detected in 42% of children. It was found that comparing hair analysis results within families provided additional information for social and legal purposes to protect children at risk [35]. It is supposed other children at risk will likely be identified by the analysis of hair collected from primary caregivers and their children connected to child deaths with drugs detected. This highlights an opportunity for earlier intervention in children who may be exposed to drugs.

There are several limitations of this study. First, the single-center study design limits the external validity and generalizability of the results. Second, the small sample size within a descriptive study must be treated with caution and limited the statistical approach. Third, missing data due to the retrospective design was problematic, the non-response rate of caregivers to questions in the checklist was high, and self-reported drug use is subject to recall bias and underreporting. These factors emphasize the importance of toxicological testing in child deaths. MA detected in hair must be interpreted within its limitations too. For example, prenatal hair replacement may not be complete until 1 year postpartum; therefore, drugs detected in hair may represent exposure before and/or after birth; children’s hair is not directly comparable to adult hair until approximately 3 years old and it was not possible to estimate the chronology of drug exposure based on the accepted average growth rate of adult head hair of 1 cm/month [21, 35]. Moreover, hair may have been collected from other regions of the head with variable growth rates due to insufficient mass at the posterior vertex. Last, hair analysis in isolation cannot distinguish between systemic and environmental exposure to MA; that said, MA detected in the hair of children necessitates further investigations to determine its significance [19–21]. Future studies to better characterize the nature of MA exposure in children might involve the hair analysis of caregivers and other children, environmental swab testing, and health assessments to determine the long-term adverse health effects, and methods to differentiate systemic and environmental exposure like exclusively endogenously formed metabolites of MA in hair.

## Conclusion

This study suggests child deaths in which MA was detected increased over the past decade. MA was reported in hair more often than in blood (94% and 18%, respectively), and it is reasonable to conclude that the greater supply of crystal MA in Australia has resulted in an increased exposure of children to MA. Case information may be inadequate to identify children at risk, and positive findings of illicit drugs in hair may have implications for other children within the same drug exposure environment.

## Key points

1. The number of child deaths in which MAwas detected has increased over the past 10 years
2. Only about one in two deceased children was known to CPS at the time of their death
3. Hair analysis was useful to identify these children, and may have implications for other children in the same drug exposure environment
